# Complete Coding Sequence of *Andean Potato Mottle Virus* from a 40-Year-Old Sample from Peru

**DOI:** 10.1128/MRA.00871-19

**Published:** 2019-10-03

**Authors:** Ian P. Adams, Cesar Fribourg, Adrian Fox, Neil Boonham, Roger A. C. Jones

**Affiliations:** a Fera Science Ltd., York, United Kingdom; b Institute for Agri-Food Research and Innovation, Newcastle University, Newcastle upon Tyne, United Kingdom; c Departamento de Fitopatologia, Universidad Nacional Agraria, Lima, Peru; d Institute of Agriculture, Faculty of Science, University of Western Australia, Crawley, Western Australia, Australia; e Department of Primary Industries and Regional Development, South Perth, Western Australia, Australia; Portland State University

## Abstract

A complete coding sequence of the type strain of *Andean potato mottle virus* from Peru (isolate Lm) was obtained. Comparison of its RNA1 and RNA2 sequences with variants of this virus isolated in Brazil revealed RNA1 and RNA2 nucleotide identities of 81 to 83% and 70 to 71%, respectively.

## ANNOUNCEMENT

In 1972, virus isolate Lm was obtained from a potato plant showing leaf mottle growing at La Molina in Lima, Peru. It constitutes the type strain of *Andean potato mottle virus* (APMoV; genus *Comovirus*, family *Secoviridae*) (1, 2). It had ∼28-nm-diameter spherical particles, was distantly serologically related to three other comoviruses, and was transmitted via contact and tubers. Its experimental host range was limited to *Solanaceae*. Infected potato plants have normally developed leaf mottle (1, 2). Another study reported two additional APMoV isolates from Peruvian potatoes. In addition to *Solanaceae*, both infected Gomphrena globosa (*Amaranthaceae*), and one infected Tetragonia expansa (*Aizoaceae*) (3). Later, APMoV infecting potato and eggplant in Brazil (4, 5) and pepper in Honduras (6) was reported. In 1976, Lm-infected leaf samples were dried over silica gel. In 1978, they were sent to Europe, becoming part of the Fera Science Ltd. (York, UK) virus collection. Although partial sequences of Brazilian isolates (7, 8) and partial coat protein (CP) and polymerase (Pol) sequences of a Chinese isolate are available in GenBank (under accession numbers KJ746645 and KJ746620, respectively), complete APMoV sequences are lacking.

Sequencing methods were as previously described (9–11). Briefly, in 2015, total RNA was extracted from preserved Lm-infected leaves using an RNAeasy kit (Qiagen, UK). From this, an indexed plant ribosome-subtracted sequencing library was constructed using a ScriptSeq complete plant leaf kit (Illumina, USA). This library was sequenced on a MiSeq instrument (Illumina) using a 600-cycle V3 kit. Reads were trimmed using Sickle 1.33 in paired-end mode set to 3′ trim paired reads to a quality score of 20 (12). A total of 137,079 read pairs were assembled using Trinity 2.0.6 at a maximum memory allocation of 99 random access memory (RAM) gigabytes with 64 central processing units allocated (13). BLAST+ 2.2.31 and the GenBank nonredundant (nr) and nucleotide databases (downloaded December 2014) were utilized to characterize the contigs (14). Contigs of viral origin were selected utilizing the extract reads function in MEGAN community edition 6.10.2 (15). Two contigs, 6,125 and 3,465 nucleotides (nt) long, were assembled and, by comparison to other comovirus genomes, represented APMoV’s RNA1 and RNA2 complete coding sequences. They were assembled from 13,028 read pairs (10% of total reads) and had 41% and 40 to 41% GC contents, respectively. Their average coverages were 481× (RNA1) and 601× (RNA2).

Lm’s RNA1 sequence had 81 to 83% nucleotide identities with partial RNA1 sequences of Brazilian APMoV isolates (7, 8), whereas its RNA2 had 70 to 71% nucleotide identities with their complete coding or partial RNA2s. Open reading frames were identified using CLC Main Workbench 6.9.1 (Qiagen, UK) and comparison with related *Comovirus* sequences. Amino acid sequence comparisons with Brazilian APMoV isolates showed that Lm’s combined CPs (small and large CP sequences) had 68% amino acid identity with these sequences, whereas their Pro-Pol had 94% amino acid identity. In sequence comparisons between Lm and partial Chinese APMoV isolates’ CP and Pol, amino acid identities were 74% (CP) and 96% (Pol). The nearest comoviruses were *Squash mosaic virus* (42% CP identity) and *Turnip ringspot virus* (45% Pro-Pol identity). The latest *Secoviridae* International Committee on Taxonomy of Viruses (ICTV) report (16) states that where the percent identity in one or both sequences is near the proposed cutoff (75 to 85% Pro-Pol or 70 to 80% CP[s]), biological properties are useful species demarcation criteria. Based on the 68% combined CP amino acid identity between Lm and Brazilian isolates, the latter isolates could be from a different species. However, because of smaller Pro-Pol amino acid identity differences, insufficient biological studies, and intermediate partial Chinese sequences, we retain the name APMoV here pending further studies. Since APMoV is a quarantine species for the European Union (EU) (EU1/A1, European and Mediterranean Plant Protection Organization [EPPO] A1), United States, and 15 other countries (17; https://gd.eppo.int/taxon/AVBO00), such studies are needed to ascertain the plant health status of its divergent isolates.

### Data availability.

The APMoV genome sequences were deposited in GenBank under the accession numbers MN176101 (RNA1) and MN176102 (RNA2). In the SRA, raw data were deposited under BioSample number SAMN12259772 and BioProject number PRJNA491634.
